# Obstacles during training of pregnant doctors in Germany before and after COVID-19: is there a need for change?

**DOI:** 10.1515/iss-2024-0021

**Published:** 2024-12-13

**Authors:** Caroline Fortmann, Susanne Johna, Christiane Groß, Maya Niethard, Barbara Puhahn-Schmeiser, and Maternity Protection Task Force

**Affiliations:** Department of Pediatric Surgery, 9177 Hannover Medical School, Hannover, Germany; Marburger Bund – Federal Association, Berlin, Germany; German Medical Women’s Association, Berlin, Germany; Department of Surgical Oncology, Helios Klinikum Berlin-Buch, Berlin, Germany; Department of Orthopedic Surgery, University Medicine Greifswald, Greifswald, Germany; Department of Neurosurgery, University Medical Center, Albert-Ludwigs-University, Freiburg im Breisgau, Germany

**Keywords:** pregnant doctors, training during pregnancy, disadvantage in pregnancy, operate during pregnancy, COVID-19 pandemic, Maternity Protection Act

## Abstract

**Objectives:**

With the growing proportion of female practitioners in the medical field, management of pregnant medical doctors is an increasingly important concern. The 2018 amendments to the German Maternity Protection Act stipulate that pregnant doctors and students can and should be permitted to safely continue their work under strict protective measures. Despite these measures, the reality is that pregnancy still results in limits on professional advancement and attainment for many physicians. To improve this situation, we analyzed the current situation in Germany. Based on our analysis, we identified some areas in need of improvement and offered recommendations.

**Methods:**

We performed a nationwide online survey that was sent to medical doctors and students via the physicians’ union Marburger Bund members’ mailing list. The survey’s main focus was on which kinds of clinical duties the pregnant doctors were assigned to, subdivided into four different time periods (under the previous and amended Maternity Protection Act, during COVID-19 pandemic and after), from 2016 to 2022.

**Results:**

Our survey included 4,748 female medical doctors and students, with a balanced distribution across the four different time periods. The most striking restriction reported by our participants was a ban on performing surgeries and invasive procedures, an experience reported by 57–65 % of pregnant doctors and medical students before the COVID-19 pandemic and nearly 80 % since the pandemic. In addition, since the COVID-19 pandemic, a complete employment ban has been enforced upon nearly 50 % of the pregnant doctors. More than 50 % of pregnant doctors considered their careers to have been obstructed or curtailed by their pregnancies prior to the pandemic, with that number growing to two-thirds since the pandemic.

**Conclusions:**

Pregnancy negatively impacts doctors’ training and careers. Many women are not allowed to perform surgeries and invasive procedures, even while some of these practitioners report that they wish to and are capable of continuing their work. Change is needed to support enthusiastic young female doctors and students and enable them to maintain their skills and professional advancement during pregnancy.

## Introduction

The medical field is becoming more and more female. For the last 17 years, more than 60 % of the medical students in Germany have been females [1]. The time at which female medical students and doctors wish to start a family often coincides with their years of medical training or early professional practice; consequently, medical students and doctors often become pregnant during these years.

The 2018 amended Maternity Protection Act in Germany allows pregnant women to safely continue their work under strict protective measures, following an individual risk assessment by the employer [2]. Enabling continued employment of women who are willing to continue their professional activities while pregnant would diminish the adverse consequences of pregnancy on these women’s careers. Furthermore, medicine is particularly reliant on the participation of every qualified practitioner due to an on-going shortage of skilled professionals.

Working conditions for pregnant doctors vary internationally. In the United States, there is no official maternity leave policy and many surgical programs do not have special protection rules for pregnant surgeons [3]. Canada offers pregnant surgical residents a reduced workload policy mostly regarding call duties and long shifts, depending on the gestational week and the province they live in [4]. In the United Kingdom, a risk assessment is recommended, mostly resulting in stopped night on-call shifts by 30 weeks [5]. Employers in Ireland are required to undertake a risk assessment of the workplace and to offer alternative duties and restricted hours if necessary [5].

Since the beginning of 2020, the COVID-19 pandemic has changed the working conditions for everyone in the medical field. Doctors worldwide have described a loss of control over their working lives and reduced training opportunities [6]. However, the consequences for pregnant women have been even more severe. Due to infection risk, most pregnant doctors or students were immediately put under an occupational employment ban, which has not changed significantly since the declared ending of the pandemic.

With the goal of ultimately improving the situation for pregnant doctors and medical students, and for their patients, we set out to analyze the current status in this group of employees in Germany during the last 7 years, using an online survey. The main objective was to examine the possibilities and restrictions encountered by pregnant women practicing medicine, as well as pregnancy’s impact on their career.

## Methods

Under the leading of the physicians’ union Marburger Bund (MB), a cooperative initiative of members of the MB, the German Medical Women’s Association (DÄB), the initiative OPidS (Operieren in der Schwangerschaft – “Operate During Pregnancy”), the German Society for Orthopedics and Trauma (DGOU), the female surgeons’ association “die Chirurginnen e.V.,” and the VLK (Verband leitender Krankenhausärztinnen und -ärzte e.V., the association of leading doctors in hospitals) developed a nationwide online survey about the experience of working as a pregnant woman in medicine. The link to the survey on the SurveyMonkey-platform was sent to all medical doctors and medical students via the Marburger Bund members’ mailing list. One reminder was sent within a newsletter. Additionally, the link to the questionnaire was sent by mail within the other cooperative units. The survey was open between November 18, 2022 and December 18, 2022. The questionnaire included general questions regarding the participants’ medical degrees and positions as well as their medical specialties. The survey also inquired about the timing of respondents’ pregnancy announcements and the possible clinical activity during pregnancy, as well as about respondents’ assessments of the potential effect of the pregnancy on their careers. Questions were based on a previously published German survey [7] and modified; furthermore, new questions were added. All questions were discussed intensively in the maternity protection task force and checked for plausibility.

For the analysis, the answers were sorted across four different time periods of pregnancy: 2016–2017 (previous Maternity Protection Act), 2018–2019 (amended Maternity Protection Act), 2020–2021 (COVID-19 pandemic), and since 2022. The previous Maternity Protection Act severely restricted clinical work for pregnant women, leading to significant career detriment. The 2018 amended Maternity Protection Act allows pregnant (future) doctors to perform special medical activities under strict protective measures, following an individual risk assessment. Thereby, the option to continue performing surgeries or other invasive procedures is left open, provided that no medical or psychosocial reasons prohibit it. With this new opportunity, the pregnant women are exposed to less discrimination and negative career impacts. During the COVID-19 pandemic, many pregnant women were immediately put under an occupational employment ban for protective reasons connected to infection risk.

Data are reported in absolute numbers and percentages. Effects were compared between women in surgery and in internal medicine using Chi-square test. Statistical analysis was performed using SPSS, version 27. p<0.05 was defined to indicate statistical significance.

## Results

**Figure 1:**
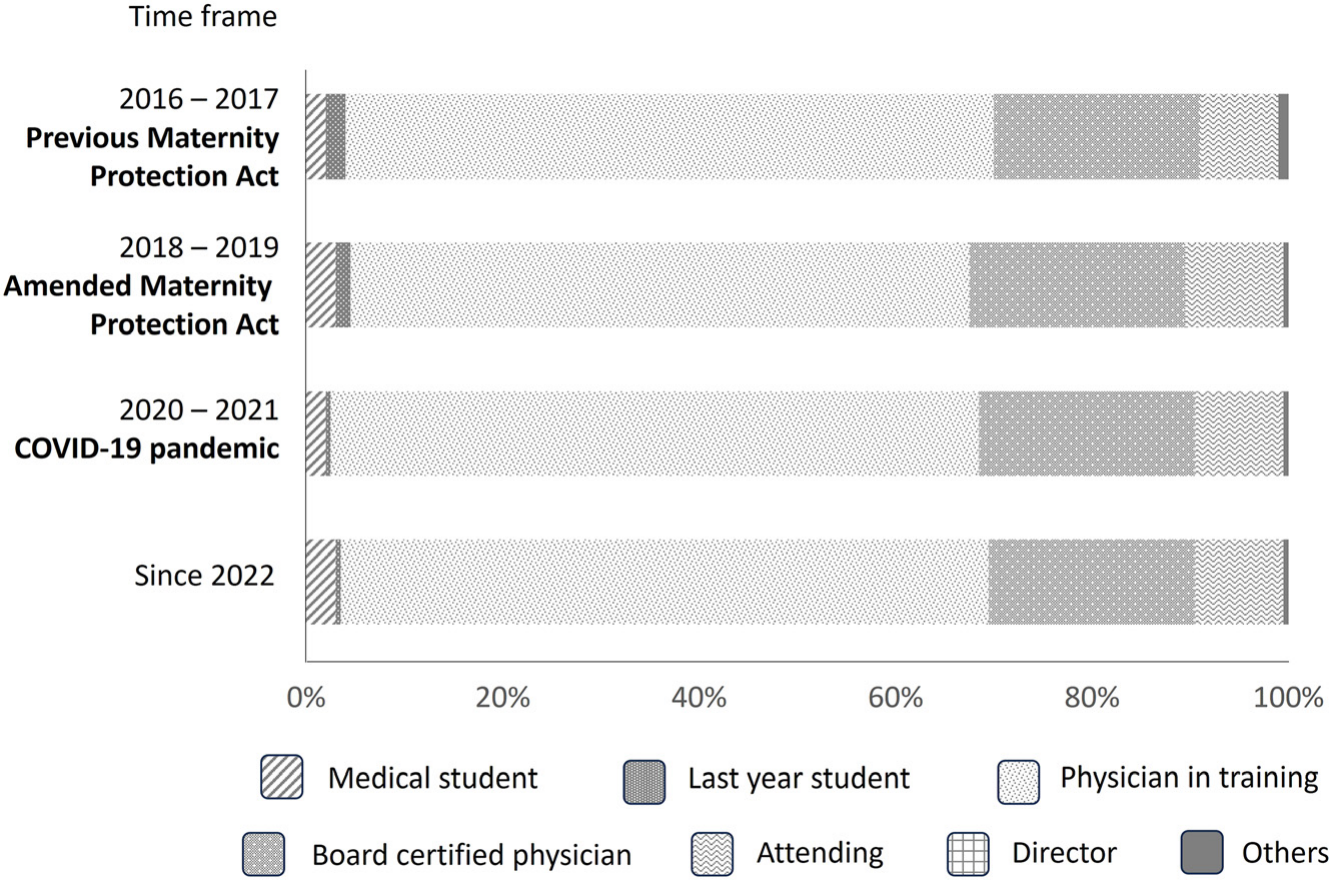
Professional position of respondents during pregnancy. Illustration of the proportion of different professional positions held by the participating women hold during their pregnancies, subdivided into the different time periods (2016–2017: previous Maternity Protection Act; 2018–2019: amended Maternity Protection Act; 2020–2021: COVID-19 pandemic; and since 2022).

During the 1-month period, 4,748 female medical doctors and female medical students from all parts of Germany participated in the online survey. The number of respondents was approximately equally distributed between the four different time periods (2016–2017: 18 %; 2018–2019: 21 %; 2020–2021: 36 %, 2022 and later: 24 %). Most of the participants (63–66 %) were undergoing their specialty training at the time of their pregnancy. The distribution was similar in the different time periods and is displayed in Figure 1. Subspecialties of participants were internal medicine (21 %), surgery (14 %), obstetrics and gynecology (10 %), general medicine (5 %), anesthesia, pediatrics, neurology, psychiatry, radiology, and others.

### Announcement of pregnancy

**Figure 2:**
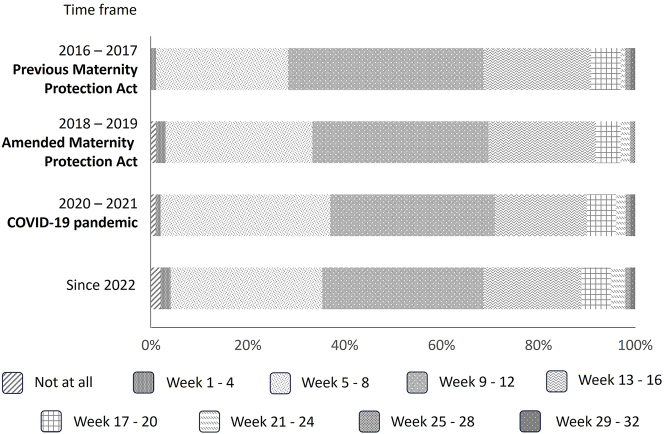
Pregnancy week at the time of the announcement of the pregnancy. Illustration showing the percentage of women who informed their respective employers about their pregnancy in each one of eight 4-weeks spans, or who did not inform their employer at all.

**Figure 3:**
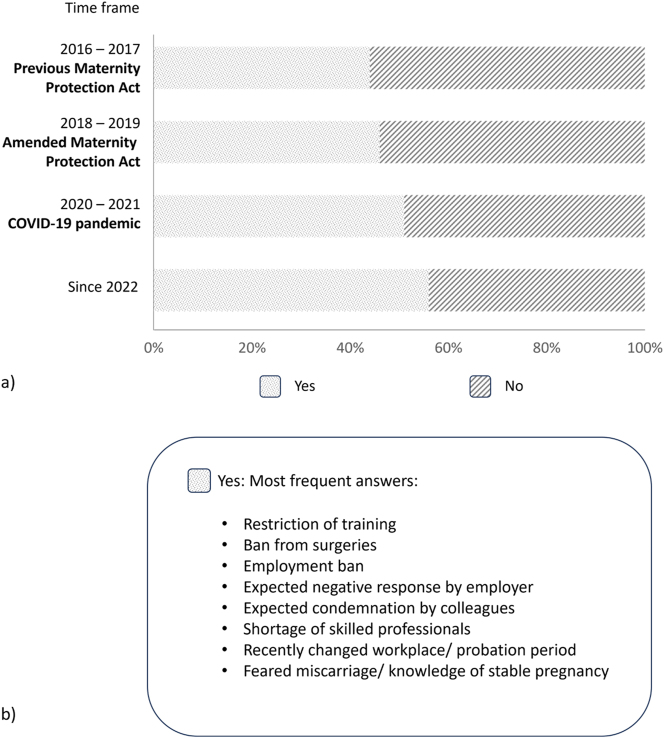
Concerns about the announcement of the pregnancy. (a) Percentages of the surveyed women expressing concern about the announcement of their pregnancy, subdivided into the different time periods. (b) Most frequently mentioned reasons for concern.

Most of the pregnant doctors and students (64–69 %) informed their employers about their pregnancies during their second and third month of pregnancy, whereas 25–28 % waited until their fourth or fifth month (Figure 2). These behaviors were fairly consistent across all of the different time periods. Reasons for the announcement were receiving the protective measures of the Maternity Protection Act, complicated pregnancy courses or knowledge of a stable pregnancy. One underlying factor for early pregnancy announcement increased over the last years; since 2022, 29 % of the doctors claimed high workload. In the previous years, these numbers were 15 % (2016/2017) and 22 % (2018/2019 and 2020/2021). Forty-four to 56 % of the doctors and students surveyed had reservations about announcing their pregnancies, mostly because of the possibility that work would be restricted, concerns about negative effects on training and career, as well as expected negative reaction on the part of directors and colleagues (Figure 3). Since 2022, the rate of women who had concerns about their pregnancy announcements increased compared to the time before the COVID-19 pandemic (56 % vs. 44–46 %). Compared to doctors in internal medicine, the rate of concerns about the announcement was significantly higher for pregnant surgeons (surgery: 355 had concerns, 262 not; internal medicine: 495 had concerns, 449 not; p=0.048).

### Risk assessment and resulting restrictions for pregnant women

**Figure 4:**
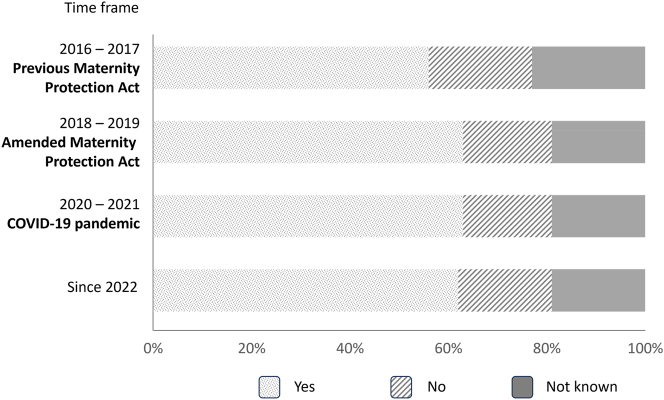
Performance of general risk assessment. Illustration of the rate of performance of a general risk assessment by the employing institution, subdivided into the different time periods.

**Figure 5:**
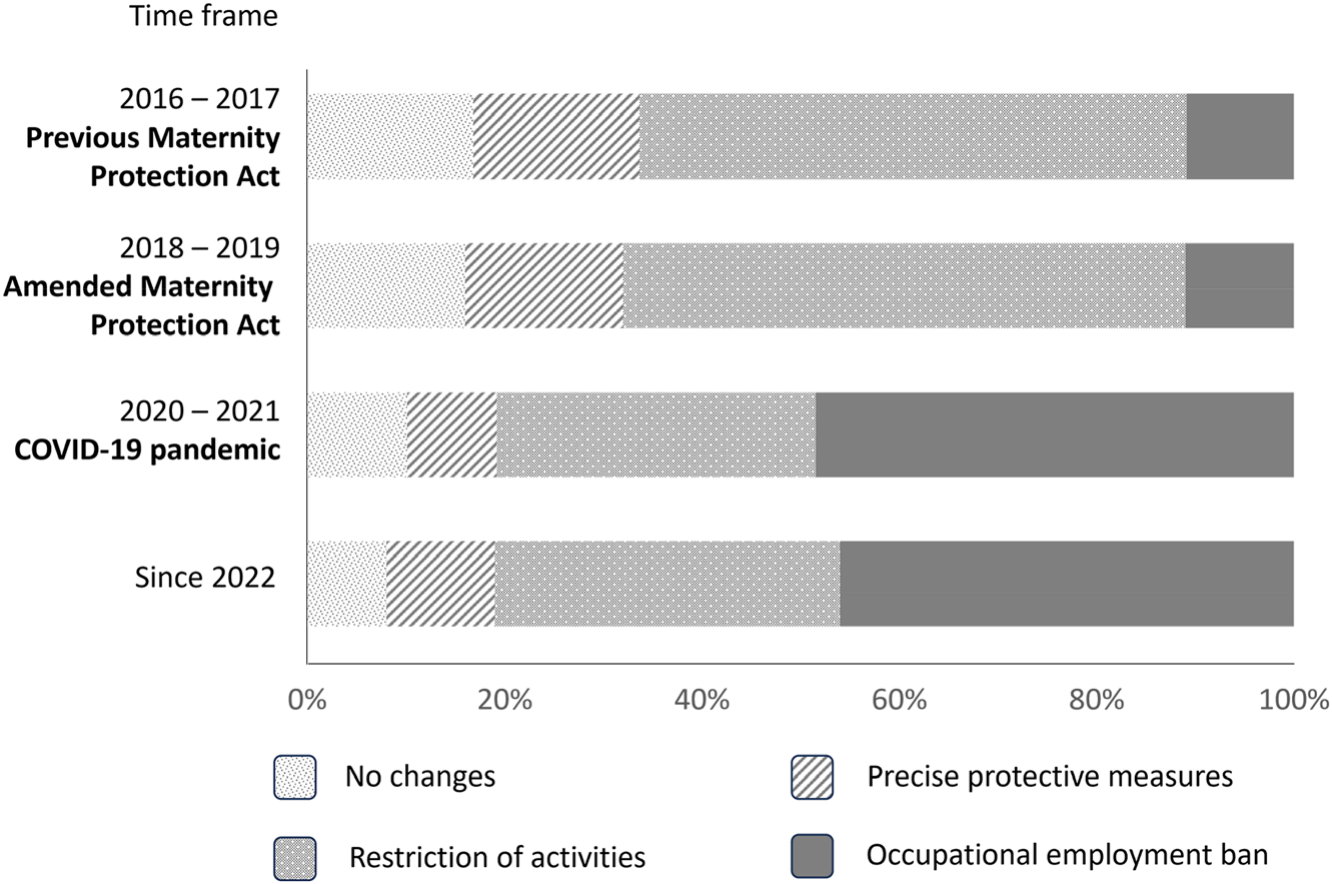
Effect of risk assessment. Illustration of the proportion of changes for the pregnant women resulting from the risk assessment, subdivided into the different time periods.

About 61 % of the doctors and students surveyed reported that their departments provided a general risk assessment. The remaining women were unaware of any performed risk assessment (Figure 4). Only 63–69 % of the pregnant women received an individual risk assessment. This resulted in restriction of previous professional activities in 56–57 % of participants before COVID-19, and 32–35 % since the COVID-19 pandemic (Figure 5). The restrictions included general protective measures resulting from the Maternity Protection Act (no night shifts, no work in the emergency department, no treatment of infectious patients). However, also a high percentage of pregnant women were not allowed to perform surgeries or other invasive activities (57–65 % before COVID-19 and 78–79 % since the COVID-19 pandemic). Seventeen percent reported no changes at work after pregnancy announcement under the previous or the amended Maternity Protection Act before the COVID-19 pandemic. After the beginning of the COVID-19 pandemic, 46–48 % of the women received a full employment ban, compared to just 11 % before the pandemic (Figure 5). These restrictions were slightly more severe in internal medicine, compared to surgery (full employment ban in 52–53 % vs. 48–51 %).

### Surgeries and invasive procedures

**Figure 6:**
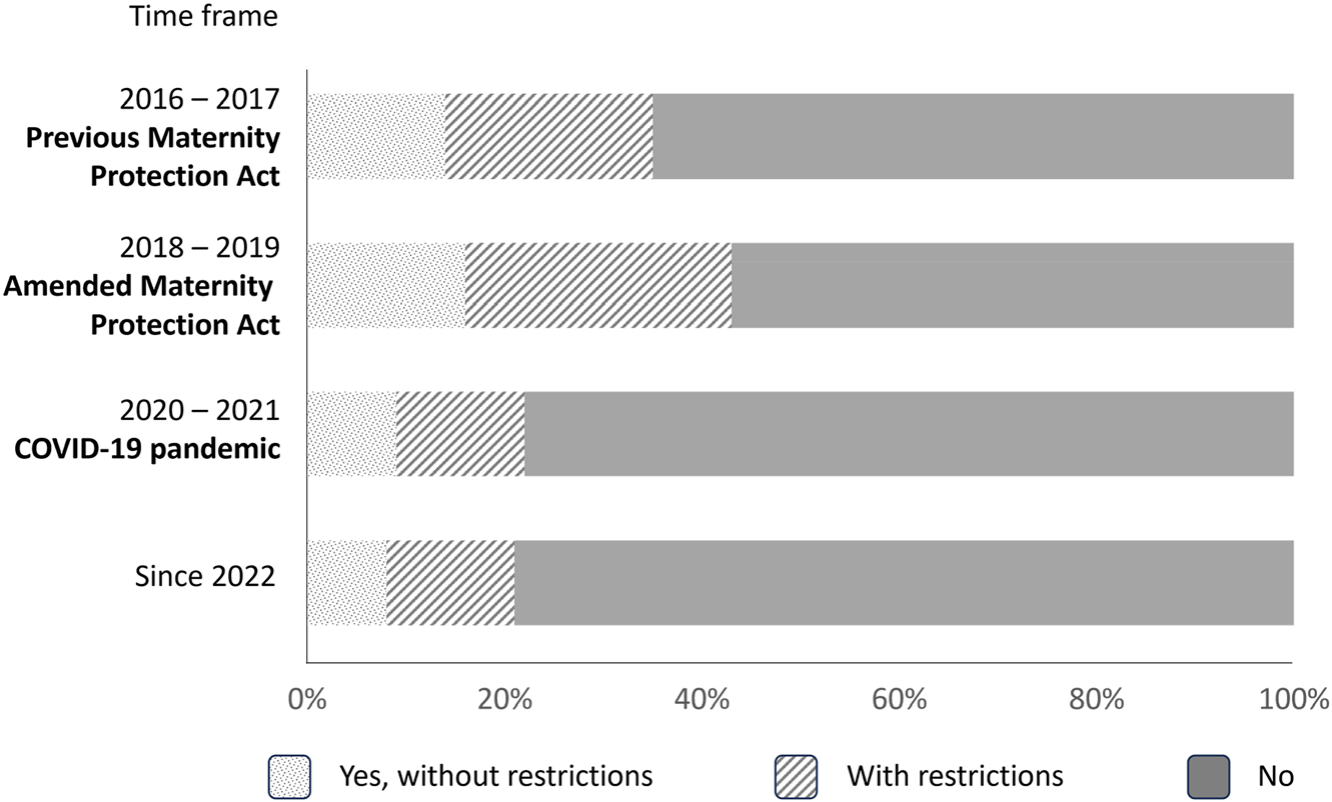
Participants’ opportunity to perform surgeries or other invasive procedures. Illustration of the proportion of participants (partially) permitted or prohibited from participation in surgeries or other invasive procedures, subdivided into the different time periods.

Surgical activities were the main restriction during pregnancy. Before the COVID-19 pandemic, more than 50 % of the pregnant (future) doctors were not allowed to perform surgeries or other invasive procedures, and this proportion has increased up to nearly 80 % since the beginning of the COVID-19 pandemic (Figure 6). Comparing the participation of pregnant women under the previous and amended Maternity Protection Act, the number of complete ban of surgeries decreased slightly from 65 to 57 % after the implementation of the amended Maternity Protection Act in 2018.

Comparison between pregnant surgeons and pregnant doctors in internal medicine showed significantly higher restrictions in internal medicine (surgery: able to perform operative procedures: 65, at a reduced rate: 104, not: 353; internal medicine: able to perform invasive procedures: 50, at a reduced rate: 92, not: 488; p<0.05).

The most often mentioned measures that allowed pregnant women to continue work were total intravenous anesthesia, surgeries without X-rays, selected planned surgeries, patient care on the ward or administrative tasks.

### Effect on training

**Figure 7:**
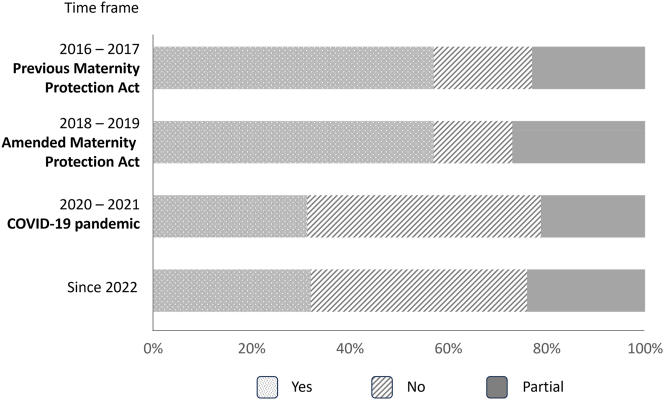
Ability to continue training. Illustration of the proportion of participants’ enabled continuance of training while pregnant, subdivided into the different time periods.

Before the pandemic, more than half of the pregnant women were able to perform training activities without restrictions. The amended maternity protection law improved the situation slightly, resulting in just 16 % of pregnant women who were unable to perform any training procedures (Figure 7). After the beginning of the COVID-19 pandemic, the negative impact of pregnancy on training was dramatic. Forty-four to 47 % of pregnant women were unable to continue their training. Training restriction was significantly higher for pregnant surgeons compared to internists (surgery: gain training contents: 110, partial: 105, not: 168; internal medicine: gain training contents: 299, partial: 157, not: 225; p<0.001).

### Effect on the career

**Figure 8:**
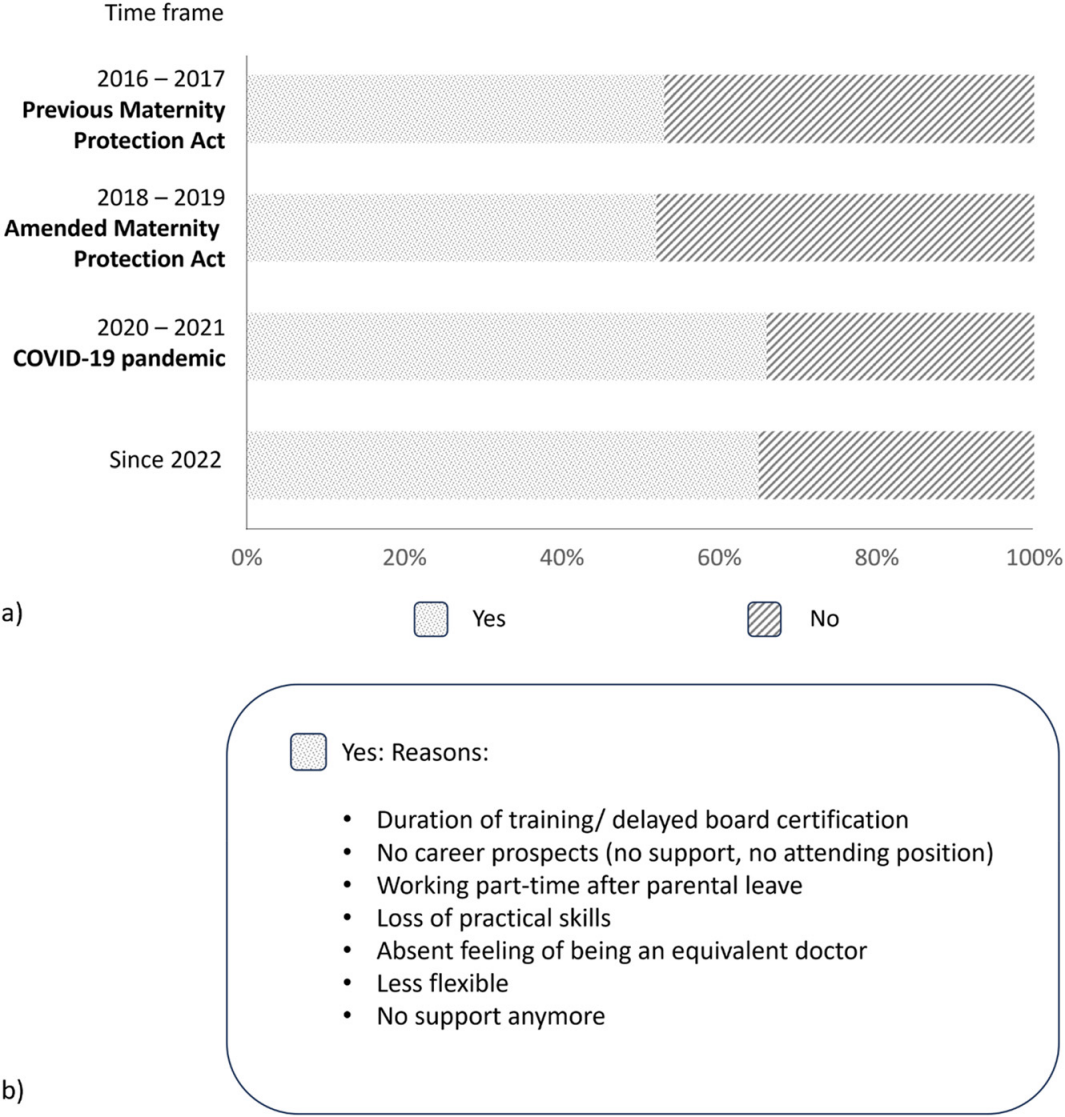
Negative effect of the pregnancy on the career. (a) Illustration of participating women reporting a negative effect of pregnancy on their career, subdivided into the different time periods. (b) Most mentioned reasons for negative effects.

Pregnancy also presented career obstacles in 52–53 % of survey participants before the pandemic, and 65–66 % after the start of the pandemic (Figure 8). Pregnant surgeons experienced significantly more frequent negative effects on the career, compared to pregnant women in internal medicine (surgery: negative effects: 409, no negative effects: 131; internal medicine: negative effects: 492, no negative effects: 352, p<0.001). Respondents mentioned different reasons: longer training periods, loss of practical skills, absent support, not being considered for attending positions, and restricted working hours after parental leave.

## Discussion

Pregnant women in medicine face an array of challenges. Our results showed that more than half of the women surveyed had concerns about the announcement of their pregnancy. While some women announced their pregnancy at a very early stage due to the high workload, in order to receive protective measures, others waited much longer in an effort to avoid restrictions on their professional activity, to delay an often-occurring impaired support and thereby a career interruption. Given the many reasons for concern faced by pregnant medical students and doctors, we can speculate why the concerns surrounding pregnancy announcements have increased notably since 2022: certainly, the high proportion of pregnant doctors in extremely high rate of restrictions of surgical and other invasive procedures since 2022 could be factors. The fear of restrictions of surgical procedures that display the main field of activities for surgeons (in and after training) could explain the significantly higher rate of concerns in surgery, compared to internal medicine.

Recent results from a nationwide survey of the situation for pregnant medical students and doctors in Germany under the 2018 amended Maternity Protection Act showed similar negative experiences and effects on the medical career of pregnant women [7]. Of the 790 participants, 43.9 % stated concerns about reporting pregnancy and 43.1 % felt set back in their careers [7]. In addition, 62.7 % reported radical change of daily work activities, while just 7.2 % continued work unaffected under strict protective measures [7].

Our results revealed that the 2018 amended Maternity Protection Act had only a minor impact, with survey participants being permitted to perform a slightly higher number of surgeries and invasive procedures compared to the years before the amendment. A survey among urologic departments in Germany in 2019 showed a similarly low trend of permitted surgeries for pregnant surgeons [8].

The COVID-19 pandemic presented a further challenge for the training and career of pregnant students and doctors. A high number of pregnant women immediately received an employment ban. The majority of pregnant women were not allowed to perform surgeries or invasive procedures anymore, effectively barring them from an essential part for their training. Pregnant doctors in internal medicine were restricted to a significantly higher degree than pregnant surgeons. Reason for this difference could be the intense work from the initiative OPidS (Operieren in der Schwangerschaft – “operate during pregnancy”), creating an advantage for pregnant surgeons. More than twice as many doctors and students reported being set back in their training due to pregnancy since the beginning of the pandemic as before its start. These results show that the COVID-19 pandemic resulted in severe negative effects on the training of pregnant (future) doctors and, therefore, their careers. This trend did not change even after implementation of the COVID-19 vaccination and increasing spread of the infection throughout the population.

Since the beginning of the COVID-19 pandemic, more than 775 million cases were reported worldwide [9]. The deaths rate of >7 million worldwide disproportionally affected older populations, not pregnant women [9], 10]. Pregnant women in the medical field needed a risk assessment; based on this, a safe environment should be created [11]. An employment ban should be the last step after changing of the working conditions or the workplace [11]. In-hospital patients were tested for the virus during the pandemic and general safety measures prohibited pregnant women to work with any infectious patients. Therefore, the infection risk was minimized, especially in the operating room. The implementation of vaccinations further improved the situation, lowered the infection rates, and reduced the risk of severe illness and death [10].

Bluntly, COVID-19 will not disappear, and the medical sector needs effective systems to safely employ pregnant women in medicine. These plans should avoid hindering their training. Protective safety measures enabling pregnant women to continue to work in the operating room already exist. When these measures are respected, the operating room is a safe environment with a manageable risk of infection.

A 2016 survey of women working in surgery showed that most female surgeons choose to operate during pregnancy because of their joy of performing surgery [12]. These results demonstrate the passion most surgeons feel for their specialty. At the same time, some other pregnant surgeons surveyed decided against a continuation of surgical activity in the operation room, mostly due to concerns about unborn life [12]. Employers should invest in creating a safe environment for pregnant doctors to enable them to safely perform surgeries if they wish to do so.

Proper risk assessment performed by the employer is also crucial to retaining trained medical manpower, and not wasting the skill and ambition of pregnant doctors eager to continue their work. Our results showed that a general risk assessment was performed in only 56–63 % of respondents’ cases, despite its requirement for every workplace. The reason for this dearth of proper assessment is unclear, but may be the result of too few employers having complete information about the necessity of the risk assessment and the enabled further work of the pregnant women.

It is well understood that discrimination against pregnant surgeons should be assiduously avoided [13]. However, many pregnant (future) doctors who want to continue working during pregnancy still face challenges. While these issues arise in other developed nations, the situation in Germany is not fully comparable to that of other countries: In the United States, for example, there are no clear standardized guidelines for pregnant doctors and no mandated paid maternity leave. Many pregnant surgeons in the United States reported having worked an unmodified schedule until delivery, including long operative hours and overnight shifts without protective safety measures, despite their own concerns about fetal and maternal health [14] and the fact that these conditions put them at increased risk for complications during pregnancy [15]. Similar results with higher complication rates than the general population were reported from a Canadian study group [16]. These findings from Northern America highlight the importance of safety measures that exist through the Maternity Protection Act in Germany, which, when followed, make it possible for pregnant women to continue their work to the extent they wish to. A recent analysis of the implementation of a parental leave policy in a general surgery residency in Colorado (USA) showed an increasing fairness feeling despite just slight maternity protection measures compared to the German Maternity Protection Act (among others no overnight in house call after 30 weeks of pregnancy and during 12 weeks after birth) [17].

The negative effect of pregnancy on training found in our survey was also reported from 61 % of general surgery residency program directors in the United States in a survey from 2016 [18]. Compared to men, a Canadian study showed that significantly more women assume that there is a negative stigma attached to pregnancy in training [16]. Another survey from the United States revealed that significantly more women in training are worried about negative career impact of parenthood than men [19]. These findings are in line with our results that 52–53 % (before the pandemic) and 65–66 % (after the pandemic) experience career obstacles due to the pregnancy/parenthood. Due to the negative effects in training and lack of supporting possibilities, many female surgeons delay pregnancy and enter family life later [15], 20], 21]. A lack of support was also described by a cross-sectional study from the United Kingdom [5]. An interesting survey from the United States showed that doctors in procedural fields were slightly older at the time of the first pregnancy than women in nonprocedural fields with no significant difference in time to conception, rates of reproductive assistance use, and pregnancy outcomes [22]. Despite significantly higher restriction rates regarding operative and other invasive procedures for doctors in internal medicine, these subspecialties seem to have enough other possibilities to gain training contents. In surgery, the restriction of surgeries seems to have a significantly higher negative effect on training and the career than restrictions in internal medicine.

The present study has several limitations. The study design relied on self-reports and subjective evaluation of similar situations is possible. Furthermore, selection bias cannot be precluded, and severe negative experiences could have motivated more women to participate than positive experiences. But the high number of respondents minimizes this possible bias.

## Conclusions

Despite liberal legislation and the clear motivation and effort to continue working in medicine in the German healthcare system, pregnancy currently presents a major obstacle for female medical doctors and students, both in their training and their career. Many pregnant women are banned from surgery and from performing invasive procedures, or even are subject to a full employment ban, even while some of these practitioners report that they wish to and are capable of continuing their work.

It is incumbent upon employers to create a safe environment, with clear protective safety measures, in support of their pregnant doctors and medical students, enabling these individuals to continue their active participation in different activities to the extent they feel comfortable. Ultimately, these protections will help to retain talented and motivated female doctors, to the benefit of the patients and the medical field as a whole.
